# Supplementary material for: Stunting at birth and associated factors among newborns delivered at the University of Gondar Comprehensive Specialized Referral Hospital
Source: PLoS One. 2021 Jan 20;16(1):e0245528. doi: 10.1371/journal.pone.0245528 (PMC7817059; doi:10.1371/journal.pone.0245528)
Supplement: S1 File — (DOC) [file pone.0245528.s001.doc]

Stunting at birth and associated factors among newborns delivered at the University of Gondar Comprehensive Specialized Referral Hospital.

Almaz Tefera Gonete^1^, Bogale Kassahun^2^, Eskedar Getie Mekonnen^3^, Wubet Worku Takele^4^ **^1, 2^**Department of Pediatrics and Child Health Nursing, School of Nursing, College of Medicine and Health Sciences, University of Gondar, Gondar, Ethiopia.

^3^Department of Reproductive and Child Health, Institute of Public Health, College of Medicine and Health Sciences, University of Gondar, Gondar, Ethiopia.

^4^Department of Community Health Nursing, School of Nursing College of Medicine and Health Sciences, University of Gondar, Gondar, Ethiopia.

**Corresponding author: Almaz Tefera Gonete**

**Email:** almazteferag3@gmail.com **Authors email adress:**

AT: almazteferag3@gmail.com

BK: bogalekassahun22@gmail.com

EGM: eskedargetie18@gmail.com

WWT: wubetakele380@gmail.com

**9.3. Annex 3: Data collection tool**

A structured and semi structured questionnaire prepared for the collection of socio-demographic, maternal, and newborn related information to assess prevalence and factors of stunting at birth among newborn-mother pairs delivered at the University of Gondar Comprehensive Specialized Referral Hospital, North West Ethiopia 2020.

**Code No. _________.**

| **Ser.**  **No.** | **Part I: Maternal Baseline Socio-demographic characteristics** | | **Skip** |
| --- | --- | --- | --- |
| 101 | How old are you? | …………………………in year |  |
| 102 | What is your religion? | 1.Orthodox   1. Muslim 2. Catholic   4.protestant  5. Others (Specify)… |  |
| 103 | Where do you live? | 1.Urban 2.Rural |  |
| 104 | What is your ethnicity? | 1. Amhara 2. Oromo 3. Tigray 4. Others (specify)......... |  |
| 105 | What is your marital status? | 1. Married 2. Single 3. Divorced 4. Widowed 5. Separated |  |
| 106 | Father’s educational status | 1.Unable to read and write |  |
|  |  | 2.read and write   1. Primary school(1-8) 2. Secondary school(9-12)   5.College and above |  |
| 107 | Mother’s educational status | 1. Unable To Read And Write 2. Read And Write   3.Primary School(1-8)  4.Secondary School (9-12)  5.College And Above |  |
| 108 | Mother’s occupational status | 1.Housewife  2.GovernmentEmployee  3.Fsarmer  4.Daily Labour  5.Merchant  6.NGO Employee  7. Other (Specify)…… |  |

**Part II. Maternal related factors**

| **No** | **Questions** | **Response** | **Remark** |
| --- | --- | --- | --- |
| 201 | Did you take iron during your pregnancy? | 1. Yes  2. No | If no skip to  Qn 3 |
| 202 | If yes? For how many days? | 1.--------------------------- |  |
| 203 | How many months have been passed just after your previous pregnancy? | 1. ----------------------------- |  |
| 204 | How many times have you been conceived (including the current pregnancy) | 1---------------------------------- |  |
| 205 | How many times did you give birth?(including the current) | 1. ------------------- |  |
| 206 | Did you have ANC follow-up during your pregnancy? | 1. Yes  2. No | If no skip to  Qn 8 |
| 207 | If yes? How frequent? | 1--------------------------------- times |  |
| 208 | Have you ever been experienced the following illnesses (select all that apply) | 1. Gestational diabetes mellitus (GDM) 2. Pregnancy-induced hypertension (PIH) 3. Others(specify__________) 4. none |  |
| 209 | Was the current pregnancy wanted? | 1. Yes  2.No |  |
| 210 | Was the current pregnancy planned? | 1. Yes  2. No |  |

**Part III: Environmental related factors**

| **No.** | **Questions** | **Responses** | |  |
| --- | --- | --- | --- | --- |
| 301. | In which season have you conceived the current pregnancy? | 1.  2. | Belg(spring)  Kiremt (rainy) |  |
|  |  | 3. | Bega (winter) |  |
|  |  | 4. | Meher (autumn) |  |
| 302. | Have you been exposed to indoor fire smoking in current pregnancy? | 1.  2. | Yes  No |  |

**Part IV: Household wealth index related characteristics**

| **No.** | **Questions** | **Response** |  |
| --- | --- | --- | --- |
| WI401 | For whom the house you live in belongs to? | 1.Private  2.Rented from individual  3.Others (specify)___________ |  |
| WI402 | How many rooms does your house have? | ____________in number |  |

| WI403 | From which material your house floor made in? | 1.Ceramic /tile  2. Cement  3 .Carpet   1. Dung 2. Earth / Sand Carpet   6.Others (specify)__________ |  |
| --- | --- | --- | --- |
| WI404 | From which material your house roof made in? | 1. Iron corrugated sheet 2. Bamboo 3. Wood 4. Thatch   Others (specify)__________ |  |
| WI405 | From which material your house’s exterior wall is made in? | 1. Stone with cement  2.Stone with mud  3. Wood with mud  4.Others (specify)__________ |  |
| WI406 | What type of fuel mainly used for household cooking? | 1. Electricity 2. Charcoal 3. Wood 4. Animal dung   5.Others(specify)___________ |  |
| WI407 | Where cooking usually done? | 1. In a separate building  Outdoors  2.In a separate room used as a  kitchen |  |

|  |  | 3.Elsewhere in the house  4.Other (*specify*)___________ | |  |
| --- | --- | --- | --- | --- |
| WI408 | Does any member of the household own any land that can be used for agriculture? | 1. Yes 2. No | |  |
| WI409 | Ownership of the farmland (if the household doesn’t have one of the two option, use) | 1.Own, in hectares/gemed____  2.Rent, in hectares/gemed____ | |  |
| WI410 | How many tones of agricultural products (includes all items) you produce annually? | ______________tone | |  |
| WI411 | Does your household have  Electricity?  A Radio?  Television?  A Non-mobile telephone?  A Refrigerator?  Table?  Chair?  A bed with cotton/spring mattress | Yes    1  1  1  1  1 | No    2  2  2  2  2 |  |
|  |  | 1 | 2 |  |
|  |  | 1 | 2 |  |
|  |  | 1 | 2 |  |
| WI412 | Does any member of your household own    A watch? | Yes | No |  |

|  | A mobile phone?    A bicycle?    A Bajaj?    Animal drawn cart?    Car? | 1  1  1  1  1  1 | 2  2  2  2  2  2 |  |
| --- | --- | --- | --- | --- |
| WI413 | Does this household own any livestock, herds, other farm animals, or poultry? | Yes  No |  |  |
| WI414 | How many of the following animals does the household have? |  |  |  |
|  | Cattle, milk cows, bulls? ( in number) | _________ |  |  |
|  | Horses, Donkeys, or mules? (in number) | _________ |  |  |
|  | Goats? (in number) | _________ |  |  |
|  | Sheep? ( in number) | _________ |  |  |
|  | Chickens? ( in number) | _________ |  |  |
|  | Beehives( in number) | _________ |  |  |
| WI415 | Does any member of this household have money in the bank Birr (ETB) if yes how much? | ---------------------- |  |  |

**Part V. Maternal anthropometric measurements**

501. Height (cm) ------------------------------------

502. MUAC (cm) ----------------------

503. Hemoglobin _________g/dl

**Part VI:** **Newborn’s sociodemographic and anthropometric measurements**

1. Sex 1. Male 2.female
2. Gestational age -------------------- (weeks)
3. Birth weight (in gram) ------------ 604. Length (cm) ----------------------
4. Birth status 1. Single birth 2. Twin 3. Multiple births

Name of data collector ____________________ sign ________ date_______ Name of supervisor ______________________ sign _________ date _______

**ነርስ ትምህርት ቤት**

**የጨቅላ ህጻናት ነርስ ትምህርት ክፍል**

*ማሳሰቢያ፡-*

የጥያቄዎቹን ክፍት ቦታዎች በትክክል መሞላቱን እና አማራጭ ያላቸዉን እንደ አስፈላጊነታቸዉ አንድ ወይም ከዚያን በላይ ምላሾች መክበብዎን አረጋግጧል ።

በመጨረሻም ከሚዘለሉት መጠይቆች በስተቀር ሁሉም መሞላታቸዉን ማረጋገጥ እና በመጠይቁ ለተሳተፉ አካላት ምስጋና አቅርቧል!!!

መጠይቁን የሚሰበስበዉ ሰዉ ስም -------------------------------------ፊርማ----------------------ቀን--------------------

የተቆጣጣሪው ሙሉ ስም------------------------------------------------ፊርማ---------------------ቀን--------------------

የመጠይቁ ኮድ ---------------------------------

| ተ.ቁ | ጥያቄዎች | አማራጭ መልሶች(መልሱን ያክቡ) | ተጨማሪ |
| --- | --- | --- | --- |
| ክፍል 1.ማህበራዊ፤ኢኮኖሚያዊ እና ስነ-ህዝባዊ መረጃን በተመለከተ ዳሰሳ | | | |
| 101 | ዕድሜ | …………/…………/……በአመት |  |
| 102 | ሃይማኖት | 1. ኦድቶዶክስ  2. ሙስሊም  3. ካቶሊክ  4. ፕሮቴስታንት  5. ሌላ ካለ(ይገለጥ) |  |
| 103 | የመኖሪያ አድራሻ | 1.ከተማ  2.ገጠር |  |
| 104 | ብሄር | 1. አማራ  2. ኦሮሞ  3. ትግሬ  4. ሌላ...... |  |
| 105 | የጋብቻ ሁኔታ | 1. ያገባች  2. ያላገባች  3. የፈታች  4. ባልየሞተባት  5. የተለያየች |  |
| 106 | የአባት የትምህርት ደረጃ | 1. ማንበብና መጻፍ የማይችል  2. ማንበብና መጻፍ የሚችል  3. የመጀመሪያ ደረጃ የጨረሰ(1-8)  4. ሁለተነኛ ደረጃ የጨረሰ(9-12)  5. ኮሌጅና ከዚያ በላይ |  |
| 107 | የእናት የትምህርት ደረጃ | 1. ማንበብና መጻፍ የማትችል  2. ማንበብና መጻፍ የምትችል  3. የመጀመሪያ ደረጃ የጨረሰች(1-8)  4. ሁለተነኛ ደረጃ የጨረሰች(9-12)  5. ኮሌጅና ከዚያ በላይ |  |
| 108 | የእናት የስራ ሁኔታ | 1. የመንግሰት ሰራተኛ  2. የቤት እመቤት  3. የቀን ሰራተኛ  4. ነጋዴ  5.መንግስታዊ ያልሆነ ድርጅት ሰራተኛ  6. ሌላ ካለ ይገለጥ... |  |

ክፍል ሁለት ከእናቶች ጋር የተዛመደ መጠይቅ

| N | ጥያቄዎች | መልስ | ተጨማሪ |
| --- | --- | --- | --- |
| 9200 | በእርግዝናዎ ወራት ኦይረን ተጠቅመው ነበር? | 1. አዎ. 2 የለም | የለም ካሉ ወደ ቁጥር 3 ይለፉ |
| 0201 | ለስንት ቀን ወስደዋል? | 1.--------------------------- |  |
| 9202 | በመጀመሪያ ልጅዎና በአሁኑ መካክል ያለው ወራት ስንት ነው? | 1. ------------------------------ |  |
| 9203 | የእርግዝና ብዛት | 1.------------------- |  |
| 2204 | የተወለዱት ህጻናት ብዛት? | 1. ------------------- |  |
| 9205 | የእርግዝና ክትትል ነበረዎት? | 1. አዎ 2. የለም | የለም ካሉ ወደ ቁጥር 7 ይለፉ |
| 0207 | ስንትጊዜ ተክታትለዋል? | 1.  2.  3.  4 |  |
| 9208 | በእርግዝናዎጊዜ ታመው ነበር? | 1.አዎ 2. የለም | ክሌለ ወደ 9 ቁጥር ይልፉ |
| Q209 | የህመምዎ አይነት ምን ነበር? | 1. በእርግዝና ጊዜ የተክሰተ የስኳር ብሸታ  2. በእርግዝና ምክኒያት የተክሰተ የደም ግፊት  3.ሌሎች  4.የለም |  |
| 2210 | በፍላጎትዎ ነበር ያረገዙት | 1. አዎ 2.አይደለም |  |
|  | እርግዝናዎ የታቀደ ነበር | 1. አዎ 2. አይደለም |  |

ክፍል አራት ከአካባቢ ጋር የተዛመደ መጠይቅ

|  | **ቅጥያቄዎች** | **መልስ** | |  |
| --- | --- | --- | --- | --- |
| 301. | የጸነሱበት ወቅት መቼ ነበር? | 1. በጋ 2. ጸደይ 3. ክረምት 4. መኸር |  |  |
|  |  |  |  |  |
| 302. | በአሁኑ እርግዝናዎ ጊዜ ለቤት ውስጥ ጭስ ተጋልጠው ነበር? | 1.  2. | አዎ  የለም |  |

**ክፍል አራት የሀብት መረጃ ጠቋሚ ጥያቄዎች**

| WI401. | የሚኖሩበት ቤት ባለቤትነቱ የማን ነው? | 1. የግሌ 2. የክራይ 3. ሌከላ ካለ ይጥቀሱ--------- |
| --- | --- | --- |
| WI402. | የሚኖሩበት ቤት ስንት ክፍል አለው? | ------------------በቁጥር |
| WI403. | የመኖሪያ ቤትዎ ወለሉ ከምን የተሰራ ነው? | 1. ሴራሚክ 2. ከስሚንቶ 3. ስጋጃ ምንጣፍ 4. በበት የተለቀለቀ 5. አፈር/አሸዋ 6. ሌላ ካለ ይጥቀሱ |
| WI404. | የመኖሪያ ቤትዎ ጣርያው ከምን የተሰራ ነው? | 1. ከብረት/ቆርቆሮ 2. ከሸንበቆ 3. ከእንጨት 4. የሳር ክዳን 5. ሌላ ካለ ይጥቀሱ---------- |
| WI405. | የመኖሪያ ቤትዎ ውጨኛው ግድግዳ ከምን የተሰራ ነው? | 1. ከድንጋይ እና ስሚንቶ 2. ከድንጋይ እና ጨቃ 3. እነጨት እና ጨቃ 4. ሌላ ካለ ይጥቀሱ--------- |
| WI406. | ከቤት ምግብ ለማብሰል ሚጠቀሙት ምንድን ነው? | 1. ኤሌክትሪክ 2. ከሰል 3. እንጨት 4. የከብት ፍግ 5. ሌላ ካለ ይጥቀሱ---------- |
| WI407. | ምግብ ሚያበስሉበት ቦታ የት ነው? | 1. የራሱ የሆነ ጨስ ቤት 2. ማድ ቤት(ቤት ውስጥ) 3. ቤት ውስጥ የትም ቦታ 4. ሌላ ካለ ይጥቀሱ--------- |
| WI408. | ምን ያህል ቃዳ መሬት አለዎት? | ______________ |
| WI409. | በአመት ውስጥ ከጠቅላላ የእህል አይነቶች ምን ያህል ኩንታል ያመርታሉ | __________ኩንታል |
| WI410. | ከሚከተሉት ውስጥ ቤትዎ ውስጥ ያለውን ሁሉ ይጥቀሱ   1. መብራት 2. ራዲዮ 3. ቴሌቪዠን 4. ቤት ስልክ 5. ፍሪጅ 6. ጠረጴዛ 7. ወንበር 8. አልጋ ከስፖንጅ ፍራሽ ጋር | አዎ የለም    1 2  1 2  1 2  1 2  1 2  1 2  1 2  1 2 |
| WI411. | ከሚከተሉት ውስጥ ቤትዎ ውስጥ ያለውን ሁሉ ይጥቀሱ   1. ሰአት 2. ሞባይል 3. የቤት ስልክ 4. ባጃጅ 5. ጋሪ 6. መኪና | አዎ የለም    1 2  1 2  1 2  1 2  1 2  1 2 |
| WI412. | ከቤትዎ ውስጥ ከብት፣ዶሮ እና በሬ አለዎት? | 1. አዎ 2. የለኝም |
| WI413. | ለተ.ቁ. 413 መልስዎ አዎ ከሆነ በቁጥር ይጥቀሱ |  |
|  | 1. ላምና በሬ | ____________በቁጥር |
|  | 1. የጋማ ከብተ | ____________በቁጥር |
|  | 1. ፍየል | ____________በቁጥር |
|  | 1. በግ | ____________በቁጥር |
|  | 1. የዶሮ ጫጩት | ____________በቁጥር |
|  | 1. የንብ ቀፎ | ____________በቁጥር |
| WI414. | ከባንክ ቤት ስንት ብር አለዎት? | ____________ብር |

**ክፍል አምስት የእናት ና የጨቅላ** ህጻን አንትሮፖሜትሪክ ልኬት

501.የእናት ቁመት በሴንቲ ሜትር-----------

502. የእናት ክንድ ዙሪያ ስፋት በሴንቲ ሜትር---------------

503. የቀይ ደም ህዋስ ልኬታ------------------

ክፍል ስድስት የጨቅላ ህጻን ማህበራዊ እና አንትሮፖሜትሪክ ልኬት

601.ጾታ ወንድ.....................ሴት..................

602. የልደት ሁኔታ አንድ ሁለት ከሁለት በላይ

603. የእርግዝና ጊዜ በሳምንት--------------------

604. የጨቅላ ህጻን ክብደት በ ግራም ----------------------

605. የጨቅላ ህጻን ቁመት በሴንቲ ሜትር ------------------

***እናመሰግናለን!***
